# An epidemiological overview of human infections with HxNy avian influenza in  the Western Pacific Region, 2003–2022

**DOI:** 10.5365/wpsar.2022.13.4.987

**Published:** 2022-12-19

**Authors:** Jozica Skufca, Leila Bell, J C Pal Molino, Dina Saulo, Chin-Kei Lee, Satoko Otsu, Kim Carmela Co,, May Chiew, Phetdavanh Leuangvilay, Sarika Patel, Asheena Khalakdina, Vanra Ieng, Tamano Matsui, Babatunde Olowokure, the WPRO Health Emergencies Programme Team

**Affiliations:** a World Health Organization Regional Office for the Western Pacific, Manila, Philippines.; b World Health Organization Representative Office for China, Beijing, People’s Republic of China.; c World Health Organization Representative Office for Lao PDR, Vientiane, Lao People’s Democratic Republic.; d World Health Organization Representative Office for Cambodia, Phnom Penh, Cambodia.

## Abstract

Avian influenza subtype A(HxNy) viruses are zoonotic and may occasionally infect humans through direct or indirect contact, resulting in mild to severe illness and death. Member States in the Western Pacific Region (WPR) communicate and notify the World Health Organization of any human cases of A(HxNy) through the International Health Regulations (IHR 2005) mechanism. This report includes all notifications in the WPR with illness onset dates from 1 November 2003 to 31 July 2022. During this period, there were 1972 human infections with nine different A(HxNy) subtypes notified in the WPR. Since the last report, an additional 134 human avian influenza infections were notified from 1 October 2017 to 31 July 2022. In recent years there has been a change in the primary subtypes and frequency of reports of human A(HxNy) in the region, with a reduction of A(H7N9) and A(H5N1), and conversely an increase of A(H5N6) and A(H9N2). Furthermore, three new subtypes A(H7N4), A(H10N3) and A(H3N8) notified from the People’s Republic of China were the first ever recorded globally. The public health risk from known A(HxNy) viruses remains low as there is no evidence of person-to-person transmission. However, the observed changes in A(HxNy) trends reinforce the need for effective and rapid identification to mitigate the threat of a pandemic from avian influenza if person-to-person transmission were to occur.

Avian influenza (AI) viruses are zoonotic but occasionally infect humans through direct or indirect contact with infected animals. In humans, infection ranges from mild to severe illness and death. Wild and domestic birds (poultry and captive birds) and other mammalian species play an important role in the emergence, evolution and transmission of different AI subtypes A(HxNy) to humans. The HxNy subtypes are classified based on the 18 subtypes of haemagglutinin (H1 through H18) and the 11 subtypes of neuraminidase (N1 through N11) on the viral surface. (*1*, *2*)

In the Western Pacific Region (WPR) of the World Health Organization (WHO), the strengthening of surveillance systems to identify human infections with AI, along with a coordinated, multisectoral approach under the One Health Initiative, have been priorities for many years. These actions have been guided by the Asia Pacific Strategy for Emerging Diseases and Public Health Emergencies (APSED III). (*3*) Global reporting mechanisms are well established to share information on A(HxNy) and guide risk assessment. Human A(HxNy) cases are notifiable under the International Health Regulations (IHR 2005) (*4*) and animal cases are notifiable to the World Organization for Animal Health under the Terrestrial Animal Health Code. (*5*)

In 2018, we published a report of notifications of A(HxNy) human cases in the WPR between 1 November 2003 and 30 September 2017. (*6*) Of the 1838 human infections with A(HxNy) in this report, most were with A(H7N9) (*n* = 1562, 85%) and A(H5N1) (*n* = 238, 13%), followed by A(H9N2) (*n* = 18, 1%) and A(H5N6) (*n* = 16, 1%). (*6*) This current report provides an update on human cases of A(HxNy) notified from 1 November 2003 to 31 July 2022.

## Methods

Human infections with A(HxNy) are commonly detected via sentinel surveillance systems, such as influenza-like illness, severe acute respiratory infection, and pneumonia with unknown etiology surveillance or through hospital-based surveillance. Member States of the WPR communicate and notify A(HxNy) human cases through the IHR (2005) mechanism to WHO. The WHO Western Pacific Regional Office has maintained a database of all official notifications of A(HxNy) human cases since 2003. This analysis includes all notifications in the WPR with illness onset dates from 1 November 2003 to 31 July 2022. Data on human infections with A(HxNy) subtypes were summarized by person, place and time and compared with results from the previous report. Data were analysed and figures were generated using Microsoft Excel.

## Results

From 1 November 2003 to 31 July 2022, there were 1972 human infections with nine different A(HxNy) subtypes notified to WHO from the WPR. Since the last report, (*6*) 134 additional human AI infections were notified from 1 October 2017 to 31 July 2022, including three new subtypes notified globally for the first time.

**Fig. 1:**
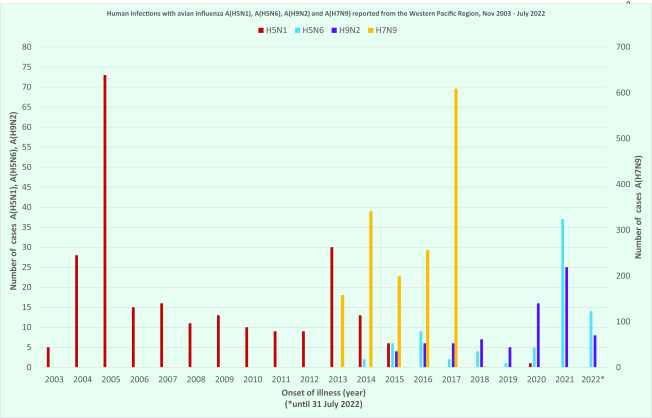
Cases of human infection with avian influenza subtypes A(H5N1), A(H5N6), A(H9N2) and A(H7N9) notified to WHO from the Western Pacific Region, 1 November 2003–31 July 2022

In the previous report, human cases with A(H5N1) and A(H7N9) were the predominant subtypes, but the majority of newly notified cases in this report were A(H5N6) (*n* = 64, 400% increase) and A(H9N2) (*n* = 59, 328% increase) (**Table 1**, **Fig. 1**).

[**insert** Figure 1]

**Table 1:** Demographic, geographic and temporal characteristics of avian influenza virus subtypes notified from the Western Pacific Region, 1 November 2003–31 July 2022

Characteristic	Influenza A virus subtype								
	H5N1	H7N9	H5N6	H9N2	H10N8	H6N1	H7N4	H10N3	H3N8
**New cases notified since last report** (*6*)**: from 1 October 2017 to 31 July 2022**									
**New cases, *n* (% increase)**	1 (< 1%)	6 (< 1%)	64 (400%)	59 (328%)	0 (0%)	0 (0%)	1 (100%)	1 (100%)	2 (100%)
**Total cases notified: from 1 November 2003 to 31 July 2022**									
**Total cases, *n***	239	1568	80	77	3	1	1	1	2
**Sex**									
Male	119 (50%)	1096 (70%)	44 (55%)	27 (35%)	1 (33%)	0	0	1 (100%)	2 (100%)
Female	120 (50%)	472 (30%)	36 (45%)	50 (65%)	2 (67%)	1 (100%)	1 (100%)	0	0
**Age, median years (range)**	20 (< 1–81)	57 (0–91)	50 (1–81)	5 (< 1–78)	73 (55–75)	20	68	41	4.5 (4–5)
Male	23 (< 1–81)	58 (1–91)	51 (3–79)	3 (< 1–39)	75	ND	ND	41	4.5 (4–5)
Female	18 (< 1–75)	56 (0–85)	47 (1–81)	5 (< 1–78)	55, 73	20	68	ND	ND
**Severity**									
Unknown	ND	142 (9%)	0	1 (1%)	0	0	0	0	0
Mild/stable	ND	89 (6%)	3 (4%)	71 (93%)	0	0	0	0	1 (50%)
Severe	ND	721 (46%)	44 (55%)	3 (4%)	3 (100%)	1 (100%)	1 (100%)	1 (100%)	1 (50%)
Deaths, *n* (CFR%)	134 (56%)	616 (39%)	33 (41%)	2 (3%)	2 (67%)	0	0	0	0
**Deaths median age (range), years**	19 (< 1–69)	60 (3–91)	48 (3–81)	48 (39–57)	74 (73–75)	ND	ND	ND	ND
**Exposure to poultry/wild birds**									
Yes	146 (61%)	741 (47%)	74 (92%)	58 (75%)	3 (100%)	0	1 (100%)	0	2 (100%)
No	7 (3%)	34 (2%)	3 (4%)	10 (13%)	0	1 (100%)	0	1 (100%)	0
Unknown	86 (36%)	793 (51%)	3 (4%)	9 (12%)	0	0	0	0	0
**Countries (% of all cases reported)**	Cambodia (*n* = 56, 23%), China (*n* = 53, 22%), Lao People's Democratic Republic PDR (*n* = 3, 1%), Viet Nam (*n* = 127, 53%)	China (*n* = 1565, 99.8%), cases with travel to China from Canada (*n* = 2), Malaysia (*n* = 1)	China (*n* = 79, 99%), Lao People's Democratic Republic PDR (*n* = 1, 1%)	China including China, Hong Kong Special Administrative Region SAR (*n* = 75, 97%), Cambodia (*n* = 2, 3%)	China (*n* = 3, 100%)	China (*n* = 1, 100%)	China (*n* = 1, 100%)	China (*n* = 1, 100%)	China (*n* = 2, 100%)
**Last reported to WHO**	31 October 2020	5 April 2019	13 June 2022	24 June 2022	13 February 2014	May 2013	14 February 2018	31 May 2021	17 May 2022

China: People’s Republic of China; CFR: case fatality rate; ND: not determined; PDR: People’s Democratic Republic; SAR: Special Administrative Region; WHO: World Health Organization.

### Human infection with A(H5N6) virus

Since the last report, an additional 64 A(H5N6) cases were reported from the WPR – 63 from the People’s Republic of China and one from the Lao People's Democratic Republic. From 2014, when the first A(H5N6) human case was notified, to 31 July 2022, 80 laboratory-confirmed cases were reported from the WPR. In 2021, a small cluster of two cases was reported in a husband and wife.

Of the 80 cases, 44 (55%) were males with ages ranging from 3 to 79 years (median 51 years) and 36 (45%) were females with ages ranging from 1 to 81 years (median 47 years). Overall, 44 (55%) were severe infections and 33 were reported to have died at the time of notification for a case fatality rate (CFR) of 41%. Most (74 cases, 93%) were exposed to wild birds or backyard poultry before illness onset (**Table 1**). The number of cases varied from two to nine per year during 2014–2020, but then increased to 37 cases in 2021. Up to the end of July 2022, 14 cases were observed (**Fig. 1**).

All but one of the cases (99%) were notified from China, across 15 different provinces. Cases were reported from Anhui (*n* = 2), Beijing (*n* = 1), Chongqing (*n* = 3), Fujian (*n* = 2), Guangdong (*n* = 14), Guangxi (*n* = 18), Guizhou (*n* = 1), Henan (*n* = 1), Hubei (*n* = 1), Hunan (*n* = 13), Jiangsu (*n* = 5), Jiangxi (*n* = 2), Sichuan (*n* = 12), Yunnan (*n* = 2) and Zhejiang (*n* = 2). In March 2021, the Lao People's Democratic Republic notified the Western Pacific Regional Office of A(H5N6) virus infection in a child identified through sentinel surveillance.

### Human infection with A(H9N2) virus

Since the last report, an additional 59 A(H9N2) cases have been notified to WHO from the WPR (57 from China and two from Cambodia). Between 2015, when the first A(H9N2) human case was notified to WHO, and 31 July 2022, 77 laboratory-confirmed cases (including two deaths, CFR: 3%), were notified from the WPR.

There were no family clusters reported, although most cases were children. Of the total cases (*n* = 77), 27 (35%) were males. Ages ranged from < 1 to 39 years (median 3 years) in males and from < 1 to 78 years (median 5 years) in females. Overall, 71 (92%) were mild infections and 58 (75%) were exposed to bird markets or backyard poultry before illness onset (**Table 1**). The number of cases observed varied from four to seven per year during 2015–2019, and increased to 16 cases in 2020, 25 cases in 2021 and eight cases up to the end of July in 2022 (**Fig. 1**).

Of the total cases, 75 (97%) were notified from China, across 16 different provinces including one from China Hong Kong Special Administrative Region (SAR). Cases were reported from Anhui (*n* = 9), Beijing (*n* = 2), Fujian (*n* = 4), Gansu (*n* = 1), Guangdong (*n* = 18), Guangxi (*n* = 3), Guizhou (*n* = 5), Henan (*n* = 2), Hubei (*n* = 5), Hunan (*n* = 11), Jiangsu (*n* = 3), Jiangxi (*n* = 2), Shandong (*n* = 1), Shanxi (*n* = 1), Sichuan (*n* = 5), Yunnan (*n* = 2) and Hong Kong Special Administrative Region SAR (*n* = 1). Two cases were notified from Cambodia in March 2021 and March 2022, both of which were in children (a 13-month-old girl and a 3-year-old boy) from Siem Reap province with mild symptoms.

### Human infection with new A(H7N4), A(H10N3) and A(H3N8)

The three different AI subtypes documented for the first time globally were from China in 2018, 2021 and 2022 comprising one case of A(H7N4), one case of A(H10N3) and two cases of A(H3N8), respectively. All cases recovered after being hospitalized and no close contacts of the cases developed illness.

The case of A(H7N4) was a 68-year-old woman with reported comorbidities and a history of exposure to live poultry before becoming ill. The case of A(H10N3) was a 41-year-old male with no clear history of exposure to poultry before illness onset. A(H10N3) was not detected in environmental samples or poultry within the locality of the case. One case of A(H3N8) was a 4-year-old boy with exposure to live backyard chickens before illness onset and the other case was a 5-year-old boy with exposure to a live poultry market (**Table 1**).

## Discussion

In recent years, there has been a change in the primary subtypes and frequency of reports of human A(HxNy) in the WPR, with a reduction of A(H7N9) and A(H5N1), and an increase of A(H5N6) and A(H9N2). Furthermore, new subtypes A(H7N4), A(H10N3) and A(H3N8) were reported from China.

The majority of cases and new A(HxNy) subtypes in the WPR were reported from China. This is likely due to several factors, including the fact that it is the world’s largest agricultural country; has an extensive human–animal interface with about 30% of the poultry raised in backyard conditions; and that live poultry markets common in China play a major part in sustaining influenza viruses as well as allowing for new reassortments of A(HxNy). (*7*-*10*) However, it could also be an indicator of strong detection, surveillance, case reporting and effective cooperation between different sectors in China. This is exemplified by the decrease in the number of human cases of A(H7N9) since 2018 owing to the united and collaborative response of multiple relevant stakeholders following a One Health approach. (*7*) Similarly, Cambodia (*11*) and the Lao People's Democratic Republic (*12*) demonstrated strong joint One Health investigation and collaboration to control the A(H9N2) and A(H5N6) human cases detected in 2021, respectively.

While human infections with A(H9N2) have mostly caused mild clinical disease and have been mostly among children (median age of 5 years), A(H5N6) can generally be more severe. However, although an increase of A(H5N6) cases was observed in 2021, the disease course and CFRs were comparable to previously detected A(H5N6) cases. In 2021, four newly detected H5N6 genotypes were the major causes of increased A(H5N6) infections. (*13*) The observed increase may reflect the spread of this virus in poultry, which is enzootic and circulates in poultry and birds in the region. (*14*) Surveillance of live poultry markets in China from 2014 to 2016 revealed that A(H5N6) replaced A(H5N1) as the dominant subtype in southern China, especially in ducks. (*15*) Additional mammal-adapted mutations were also detected, indicating the viral adaptation process from birds to humans. (*13*) However, although human A(H5N6) cases were reported from China from December 2021 to March 2022, no poultry/bird outbreaks of A(H5N6) were notified to the World Organization for Animal Health, which may suggest an underreporting of poultry outbreaks. (*2*)

The increase in reported human cases of A(H5N6) may also be due to enhanced diagnostic capacity for respiratory disease surveillance during the COVID-19 pandemic in the context of generally increased awareness of respiratory illness across the public health system. (*14*, *16*) While in China the majority of A(H5N6) cases were reported through pneumonia surveillance systems and identified by Chinese National Influenza Surveillance Network laboratories, 15 cases in 2020 and 2021 were first identified through third-party sequencing agencies that then reported to the Chinese National Influenza Surveillance Network laboratories for confirmation. More than one third of cases in 2021 were detected by hospitals that sent samples from patients with pneumonia to these third-party sequencing agencies. (*13*)

Since our last report, notifications of A(H5N1) infections have remained low despite enhanced surveillance, detection, awareness and reporting following the COVID-19 pandemic. This may indicate a true decline in A(H5N1) not biased by changes in surveillance. The A(H5N1) viruses detected during late 2021 and 2022 are different from earlier H5N1 bird flu viruses. Current viruses are not spreading easily among poultry, are infecting people less easily, and may be less of a risk and cause less severe illness among humans. (*17*)

In addition to the three new strains reported from China for the first time globally, the first two human cases of A(H5N1) were reported from Europe and the Americas in 2022. In January 2022, the first human A(H5N1) case was reported in the United Kingdom of Great Britain and Northern Ireland in a person who kept birds domestically. (*18*) At the beginning of May 2022, the first A(H5N1) case in the United States of America was reported in a person involved in bird-culling procedures. (*19*) These two sporadic cases were not unexpected since the circulation of AI viruses in poultry increases the risk of sporadic human infections, especially for those with occupational exposure. The first human cases of A(H5N8) were documented in seven workers who were involved in culling operations in a poultry outbreak in the Russian Federation in 2020. (*20*)

There are several limitations in the interpretation of these results as they are based on IHR (2005) notifications. First, the estimated CFRs for A(HxNy) should be interpreted with caution since these are calculated from the last update of notifications without any follow-up of severe cases that may have subsequently died. Given the lack of updated case information, the true number of deaths may also be skewed. Second, the capacity to detect A(HxNy) evolved during the COVID-19 pandemic when influenza surveillance systems were strengthened with changes in the sources of case detection such as through influenza-like illness surveillance. This detection capacity may vary by geographical location due to differences in surveillance systems. In addition, official notification of cases may be underreported, particularly for subclinical infections. A seroprevalence study conducted in Cambodia among poultry workers found an overall prevalence of 4.5% and 1.8% for antibodies against A(H5N1) and A(H9N2), respectively. Hence, the true burden of infection is likely higher than that observed. (*21*) Despite these limitations, reporting as outlined in the IHR (2005) continues to provide important information about human cases of A(HxNy) in the WPR and globally. It enables Member States to understand the epidemiological situation of human AI cases, assess the risks and take preventive public health actions.

In conclusion, the overall public health risk from known A(HxNy) viruses at the human–animal interface remains low as infections have been almost exclusively associated with contact with infected birds, with no evidence of person-to-person transmission. However, the observed changes in A(HxNy) trends reinforce the need for early detection and strengthening of human and animal surveillance to detect virological, epidemiological and clinical changes associated with circulating A(HxNy). Accordingly, continued multisectoral collaboration at the human–animal interface is needed for effective mitigation of the pandemic threat of AI.
